# Denatonium inhibits growth and induces apoptosis of airway epithelial cells through mitochondrial signaling pathways

**DOI:** 10.1186/s12931-015-0183-9

**Published:** 2015-02-05

**Authors:** Xiaoxing Wen, Jian Zhou, Dan Zhang, Jing Li, Qin Wang, Nana Feng, Haixing Zhu, Yuanlin Song, Huayin Li, Chunxue Bai

**Affiliations:** Department of Pulmonary Medicine, Research Institute of Respiratory Disease, Zhongshan Hospital, Fudan University, No. 180 Fenglin Road, Shanghai, 200032 China

**Keywords:** Denatonium, Bitter taste receptors, Epithelium injury, Mitochondria, Cytochrome c

## Abstract

**Background:**

Denatonium, a widely used bitter agonist, activates bitter taste receptors on many cell types and plays important roles in chemical release, ciliary beating and smooth muscle relaxation through intracellular Ca^2+^-dependent pathways. However, the effects of denatonium on the proliferation of airway epithelial cells and on the integrity of cellular components such as mitochondria have not been studied. In this study, we hypothesize that denatonium might induce airway epithelial cell injury by damaging mitochondria.

**Methods:**

Bright-field microscopy, cell counting kit-8 (CCK-8) assay and flow cytometry analysis were used to examine cellular morphology, proliferation and cell cycle, respectively. Transmission electron microscopy (TEM) was used to examine mitochondrial integrity. JC-1 dye and western blotting techniques were used to measure mitochondrial membrane potential and protein expression, respectively.

**Results:**

For airway epithelial cells, we observed that denatonium significantly effects cellular morphology, decreases cell proliferation and reduces the number of cells in S phase in a dose-dependent manner. TEM analysis demonstrated that denatonium causes large amplitude swelling of mitochondria, which was confirmed by the loss of mitochondrial membrane potential, the down-regulation of Bcl-2 protein and the subsequent enhancement of the mitochondrial release of cytochrome c and Smac/DIABLO after denatonium treatment.

**Conclusions:**

In this study, we demonstrated for the first time that denatonium damages mitochondria and thus induces apoptosis in airway epithelial cells.

**Electronic supplementary material:**

The online version of this article (doi:10.1186/s12931-015-0183-9) contains supplementary material, which is available to authorized users.

## Introduction

Denatonium is a widely used bitter taste agonist and has been demonstrated to activate bitter taste receptors on various cell types including taste cells [1], enteroendocrine cells [2,3], hindbrain neurons [4], airway epithelial cells [5], nasal solitary chemosensory cells [6] and airway smooth muscle cells [7]. Activation of bitter taste receptors increases intracellular Ca^2+^ levels via a G-protein-coupled receptor (GPCR) cascade involving phospholipase C (PLC)/inositol 1,4,5-trisphosphate (IP_3_) signaling [4-6]. This increase in intracellular Ca^2+^ leads to neurotransmitter release and taste detection in bitter taste receptor cells [8,9], insulin secretion by pancreatic islets [2], cholecystokinin (CCK) [10] and glucagon-like peptide-1 [11] release from enteroendocrine cells, CCK release from neurons [4], antimicrobial peptide release from nasal solitary chemosensory cells [6], and acetylcholine release from polymodal urethral chemosensory cells [12]. However, the effects of the bitter agonist denatonium on the cellular components of airway epithelial cells, such as mitochondria, have not been studied.

Mitochondria are important cellular structures that supply cellular energy by generating adenosine triphosphate (ATP). Furthermore, mitochondria act as internal calcium stores through the uptake of intracellular free Ca^2+^, and this is critical for calcium buffering [13]. When mitochondrial membranes are damaged, mitochondria lose their calcium buffering capability [13], and mitochondria-related proteins, such as cytochrome c and Smac/DIABLO, are released from the mitochondria into the cytosol, resulting in cellular apoptosis [1,14-16]. In contrast, Bcl-2 proteins can prevent apoptosis by blocking the release of cytochrome c from mitochondria [17-20]. Thus, mitochondria play significant roles in the cell cycle, cell growth, and cell death [21].

In this study, we hypothesized that denatonium induces airway epithelial cell injury by damaging mitochondria. We found that denatonium inhibits airway epithelial cell proliferation, reduces the number of cells in S phase and increases cellular apoptosis in a dose-dependent manner via a mitochondrial signaling pathway.

## Materials and Methods

### Cell culture

Airway epithelial cells (human lung cancer cell line A549 and human bronchial epithelial cell lines 16HBE and BEAS-2B) were cultured in Dulbecco’s modified Eagle medium (DMEM) (Invitrogen, Carlsbad, CA, USA) supplemented with 10% heat-inactivated fetal bovine serum and 100 U/ml penicillin/streptomycin. Cells were grown as monolayers in a humidified atmosphere containing 5% CO_2_ at 37°C. The culture medium was replaced with treatment medium containing the desired concentrations of chemicals every 24 h.

### Immunohistochemistry assay

Lung tissues were collected from 8- to 10-week-old wild-type C57BL/6 J mice (Animal Center, Fudan University, Shanghai, China) for the immunohistochemistry assay. The experimental protocol was approved by the Committee of Animal Care of Fudan University. All animals were handled in accordance with the Guideline for the Care and Use of Laboratory Animals. Briefly, lung tissues were harvested and fixed in formalin and then embedded in paraffin. The sections were incubated with primary antibodies including taste receptor type 2 member 10 (TAS2R10), guanine nucleotide-binding protein G(t) subunit alpha-3 (GNAT3), and transient receptor potential cation channel subfamily M member 5 (TRPM5) (Abcam Inc., Cambridge, MA, USA) and, then, were incubated with horse radish peroxidase-linked secondary antibody.

### Measurement of intracellular Ca^2+^ signaling

To monitor free intracellular Ca^2+^ in epithelial cells, Hank's balanced salt solution (HBSS) with 5 μM Fluo-4/AM (Invitrogen, Carlsbad, CA) was applied to A549 cells for 30 minutes at room temperature. Subsequently, the cells were kept in HBSS for another 20 minutes at room temperature before imaging. Confocal imaging was performed using a Nikon A1R confocal laser scanning microscope system (Nikon Corp., Tokyo, Japan). Fluo-4 was excited with a 488-nm laser, and fluorescence images (512 × 512 pixels) were collected. Regions of interest (ROI; 3 × 3 pixels) were selected in individual epithelial cells using ImageJ software (v. 1.42, Wayne Rasband, NIH) to track the changes in fluorescence intensity. The fluorescence intensity ratio (F/F_0_) was calculated by dividing fluorescence intensity at time t (F) with the fluorescence intensity at the beginning of the experiment (F_0_).

### Morphological examination of airway epithelial cells

To examine the morphological change in airway epithelial cells, 8 × 10^5^ cells were plated in a 60-mm tissue culture dish and were treated with denatonium benzoate (Sigma-Aldrich, St. Louis, Missouri, USA). As our preliminary results indicated that different airway epithelial cells had differing sensitivities to denatonium, 16HBE, BEAS-2B, and A549 cells were treated with denatonium for 24 h, 48 h, and 72 h, respectively, to achieve similar percentages of cell death for all three cell lines. The bright field images of the airway epithelial cells were taken with a fluorescent microscope (Olympus Corp., Tokyo, Japan) connected to a digital camera. Ten randomly chosen microscopic fields from each dish were analyzed.

### Cell proliferation and viability assay

Cell Counting Kit-8 (CCK-8; Dojindo Laboratories, Japan) was used to assess the rate of cellular proliferation and to quantify cell viability. In brief, airway epithelial cells were plated in 96-well plates at approximately 2000 cells per well with 100 μL culture medium and were treated with denatonium at different concentrations and for different lengths of time. Then, 10 μl of CCK8 solution was applied to each well, and the plates were incubated for 1 h at 37°C. Finally, the absorbance values at 450 nm were determined using a microplate reader (FLX800TBID, Biotek instruments, VT, USA). All experiments were conducted in triplicate.

### Transmission Electron Microscopy (TEM) analysis of cellular components

After a 72 h-incubation with 2 mM denatonium, the treated and non-treated control A549 cells were collected, washed with PBS and then fixed in 2.5% glutaraldehyde (PH = 7.4) at 4°C overnight. After washing with 0.1 M phosphoric acid solution, the cells were post-fixed in 1% buffered osmium tetroxide, dehydrated in graded alcohols, embedded in Epon 812, cut into 50–60 nm ultra-thin sections and stained with uranyl acetate and lead citrate. Finally, ultrathin sections were examined with a transmission electron microscope (CM120, Philips, Hillsbro, USA).

### Mitochondrial membrane potential measurement

A JC-1 mitochondrial membrane potential assay kit (Beyotime Biotechnology Inc., Nantong, China) was used to detect changes in mitochondrial membrane potential. JC-1 is a fluorescent lipophilic cationic probe. It accumulates in healthy mitochondria to form J-aggregates emitting red fluorescence at 590 nm and accumulates in depolarized mitochondria as J-monomers emitting green fluorescence at 490 nm. An increased ratio of green-fluorescent cells indicates mitochondrial damage. The assay was performed according to the kit’s instructions, and the results of the assay were obtained by flow cytometry (Accuri Cytometers, Inc., MI, USA).

### Detecting apoptosis by flow cytometry

An annexin V-FITC and propidium iodide (PI) double staining kit (Invitrogen, Carlsbad, CA, USA) was used to analyze cellular apoptosis. Airway epithelial cells were seeded into 6-well plates (5 × 10^5^ cells/well) and treated with denatonium at different concentrations and for different lengths of time. The cells were digested with trypsin (Gibco® Trypsin-EDTA, Invitrogen, Carlsbad, CA, USA), washed with PBS three times, suspended in 500 μl binding buffer and, finally, incubated with 5 μl of FITC-conjugated Annexin-V and 5 μl of PI for 15 min at room temperature in the dark. Then, the samples were analyzed by flow cytometry.

### Cell cycle analysis

Airway epithelial cells were plated in 6-well plates and treated with denatonium at different concentrations and for different lengths of time. The cells were collected, washed with PBS, and fixed in chilled 70% ethanol for 24 h at 4°C. Then the fixed cells were stained with propidium iodide (PI) staining solution (40 μg/ml RNase A and 50 μg/ml PI in PBS) in the dark for 30 min at room temperature. Finally, cell cycle distribution was analyzed by flow cytometry.

### Western blot assay

Total protein was extracted using a RIPA kit (Beyotime Biotechnology Inc., Nantong, China). Mitochondrial and cytosolic fractions were separated using a Cell Mitochondria Isolation Kit (Beyotime Biotechnology Inc., Nantong, China). The isolated proteins were separated on polyacrylamide gels and transferred to PVDF membranes. The membranes were incubated with anti-cytochrome c (Cell Signaling Technology (CST), MA, USA), anti-Smac/DIABLO (CST), anti-Cox IV (CST), anti-Bcl-2 (CST), anti-actin (CST), anti-TAS2R4 (Abcam, Cambridge, MA, USA), and anti-GNAT3 (Abcam) at 4°C overnight and were then incubated with horseradish peroxidase-conjugated goat anti-rabbit or anti-mouse immunoglobin G at room temperature for 1 hour. The proteins were visualized using Pierce ECL Western Blotting Substrate and autoradiography. The blots were analyzed using Quantity One 4.6 software.

### Statistical analysis

The data are expressed as the means ± SEM of at least three independent experiments. The statistical analysis was performed using one-way analysis of variance (ANOVA) followed by Bonferroni’s multiple comparison test. A p-value <0.05 was considered statistically significant.

## Results

### Expression of bitter taste receptors and their downstream signaling effectors

**Figure 1:**
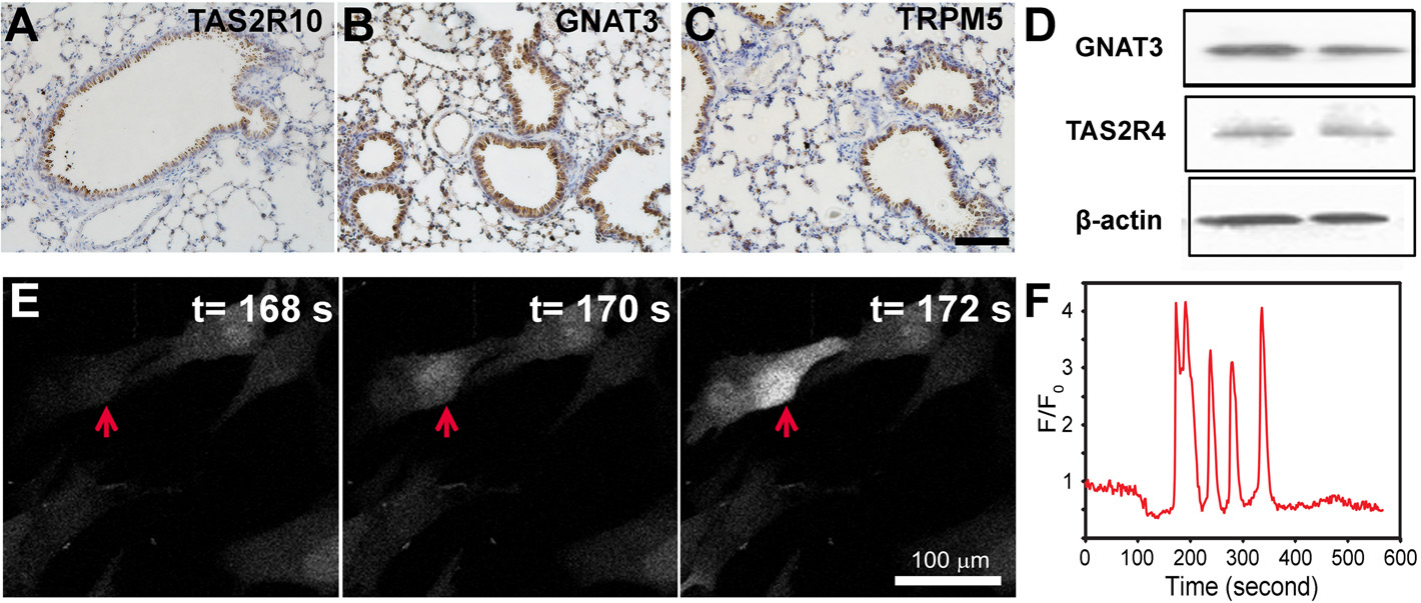
**Functional expression of bitter taste receptors and their downstream signaling effectors. A)** Immunohistochemistry images showed that bitter taste receptor TAS2R10 and its downstream signaling effectors GNAT3 **(B)** and TRPM5 **(C)** were highly expressed on airway epithelial cells in mice. Scale bar: 100 μm. **D)** Western blot showed that bitter taste receptor TAS2R4 and GNAT3 were expressed on A549 cells. **E)** I*n vitro* experiments showed that 1 mM denatonium triggered Ca^2+^ oscillations in A549 human epithelial cells. A549 cells were stained with Fluo-4 to visualize intracellular free Ca^2+^. Red arrow points to the region of interest (ROI) in an A549 cell. **F)** Ca^2+^ oscillations started immediately after denatonium application and lasted for a few cell cycles.

Immunohistochemistry studies were conducted to investigate the protein expression of bitter taste receptors and their downstream signaling effectors in airway epithelial cells. We found that the bitter taste receptor TAS2R10 and its downstream signaling effectors GNAT3 (guanine nucleotide binding protein, alpha transducing 3) and TRPM5 (transient receptor potential cation channel, subfamily M, member 5) were highly expressed on airway epithelial cells in mice (Figure 1A-C). Furthermore, we also detected the protein expression of the bitter taste receptor TAS2R4 and its downstream signaling effector GNAT3 on A549 cells (Figure 1D). The *in vitro* experiments showed that 1 mM denatonium triggered Ca^2+^ oscillations in A549 human epithelial cells (Figure 1E). The Ca^2+^ oscillations started immediately after denatonium application and lasted for a few cell cycles (Figure 1F).

### Denatonium inhibits epithelial cell proliferation and increases apoptosis

**Figure 2:**
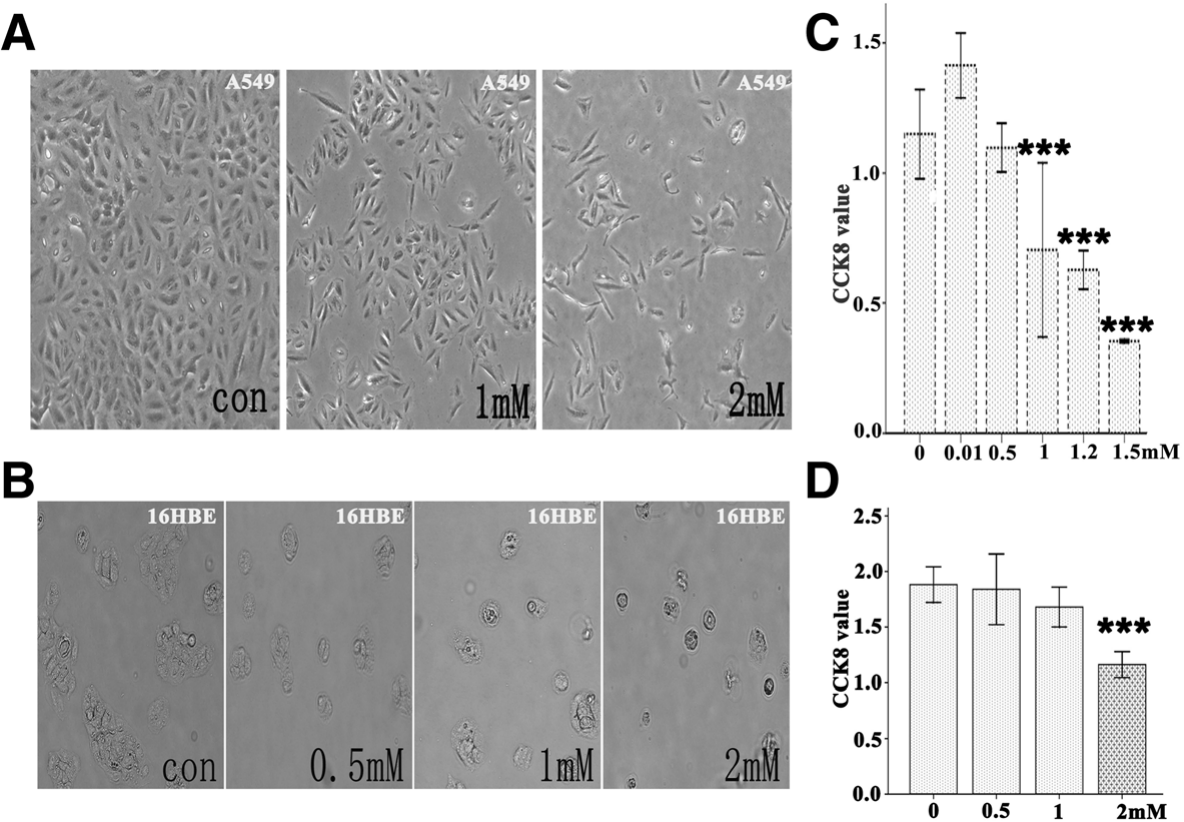
**Denatonium inhibits A549 and 16HBE cell proliferation and induces cell morphological changes. A)** Bright-field images of cultured A549 cells showed that treatment with denatonium for 72 h induced cell morphological changes. **B)** Bright-field images of cultured 16HBE cells showed that treatment with denatonium for 24 h induced cellular morphology changes. **C)** Denatonium markedly inhibited the growth of A549 cells in a dose-dependent manner. **D)** Denatonium markedly inhibited the growth of 16HBE cells in a dose-dependent manner. One representative experiment with n = 3 is shown. The error bars represent mean values ± SEM. ***indicates significant difference at *p* < 0.001 versus control.

To determine whether denatonium affects the growth of airway epithelial cells, we measured the proliferation of airway epithelial cells (A549, 16HBE, and BEAS-2B cells) treated with denatonium. Denatonium treatment induces dose-dependent cellular morphology changes. As shown in Figure 2A&B and Additional file 1: Figure S1A, untreated airway epithelial cells are densely packed, whereas airway epithelial cells treated with denatonium were rounded, shrunken, and detached from each other.

To further confirm the effects of denatonium on airway epithelial cell proliferation, the CCK-8 assay was used to assess cell proliferation and quantify cell viability. As shown in Figure 2C&D and Additional file 1: Figure S1B, denatonium markedly inhibited the growth of airway epithelial cells in a dose-dependent manner.

### Denatonium induces apoptosis of airway epithelial cells and reduces the number of airway epithelial cells in S phase

**Figure 3:**
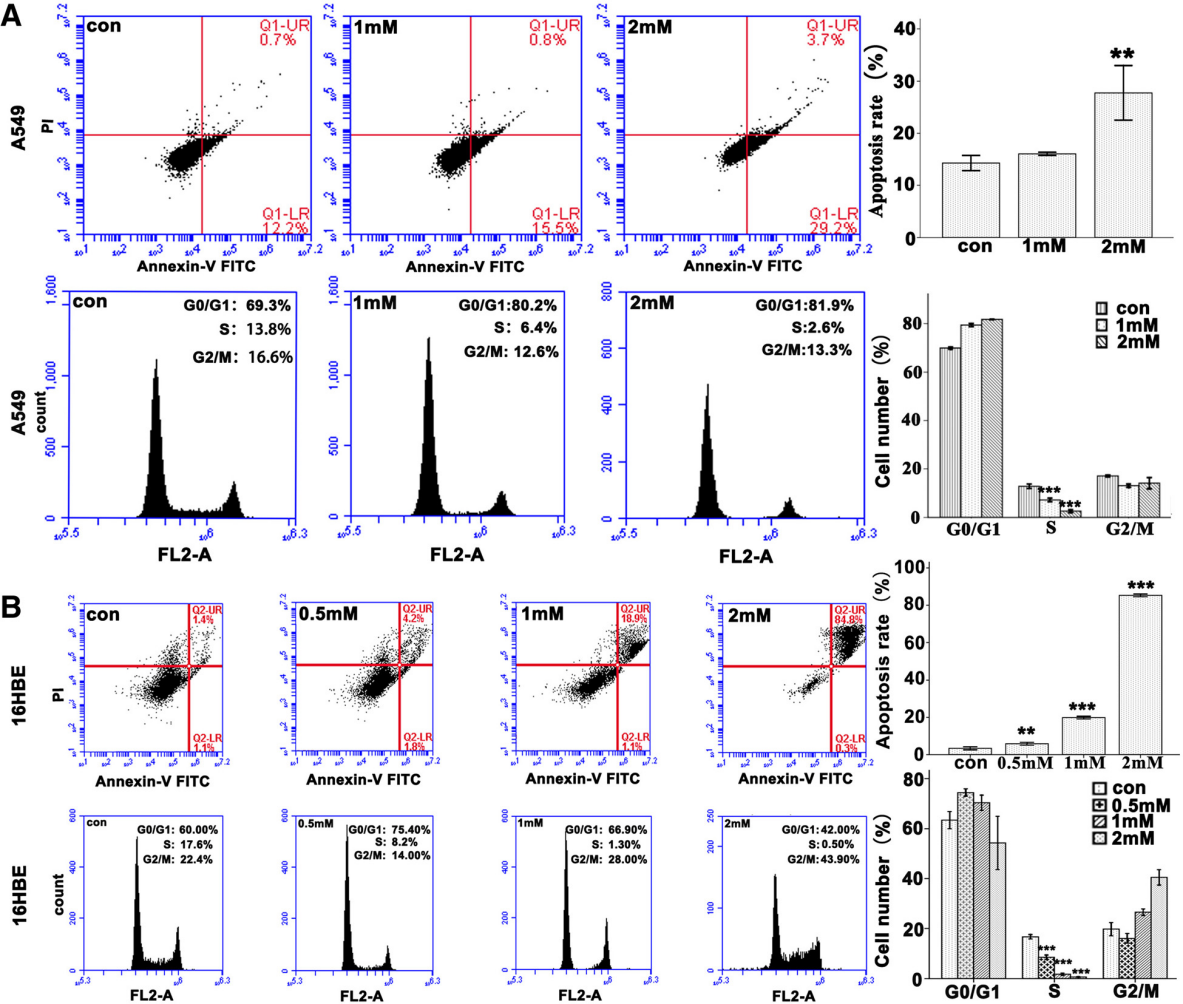
**Flow cytometric analysis of apoptosis induction and cell cycle distribution in A549 and 16HBE cells. A)** A549 cells were treated with denatonium (1 mM or 2 mM) for 72 h, stained with FITC-annexin V/PI and then analyzed by flow cytometry. The right panel shows the apoptosis rates of the cells of the various groups. Flow cytometry was also used to analyze DNA at the G1, S, and G2 phases of the cell cycle. **B)** 16HBE cells were treated with denatonium (0.5 mM, 1 mM or 2 mM) for 24 h. Then the number of apoptotic cells and the number of cells in different stages of the cell cycle were detected by flow cytometry. Data are representative of three similar experiments. **indicates significant difference at *p* < 0.01 versus control and ***indicates significant difference at *p* < 0.001 versus control.

To evaluate the effects of denatonium on airway epithelial cell apoptosis, we performed an annexin V-FITC/PI double staining assay and flow cytometry analysis. The cells in the upper-right (UR) and lower-right (LR) quadrants of the FACS histogram represent apoptotic cells. As shown in Figure 3 and Additional file 2: Figure S2, denatonium treatment of airway epithelial cells resulted in more apoptotic cells compared with no treatment. We also explored the effect of denatonium on the cell cycle of airway epithelial cells and found that 2 mM denatonium exposure caused a drastic reduction in the number of cells in S phase compared with no treatment (Figure 3 and Additional file 2: Figure S2).

### Denatonium induces mitochondrial damage

**Figure 4:**
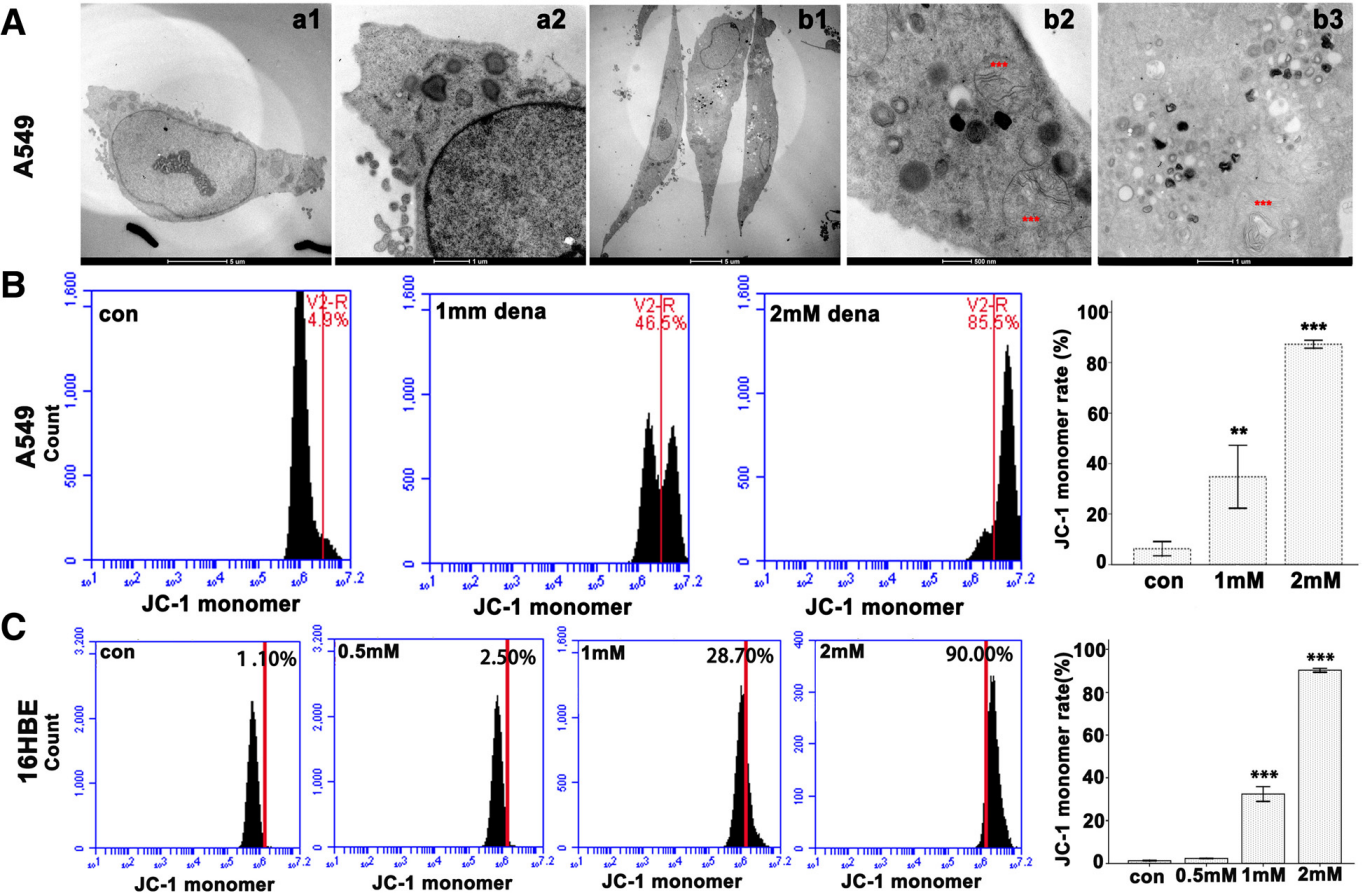
**Denatonium reduces mitochondrial membrane potential. A)** The TEM microphotographs show the ultrastructure of control cells (a1,a2) and denatonium (2 mM, 72 h) treated A549 cells (b1, b2, b3). Swollen mitochondria (***) were observed in treated cells (b2, b3). **B)** A549 cells were stained with the cationic lipophilic dye, JC-1 and analyzed by FACS after treatment with denatonium for 72 h. **C)** 16HBE cells were stained with the cationic lipophilic dye JC-1 and analyzed by FACS after treatment with denatonium for 24 h. The figures are representative profiles of at least three experiments. The histogram shows the quantification of mitochondrial membrane potential. (**p < 0.01, ***p < 0.001).

To examine whether denatonium induces mitochondrial damage in airway epithelial cells, we used transmission electron microscopy to examine the ultrastructures of A549 cells treated with denatonium. The control cells appeared to have normal mitochondria and mostly homogeneous cytoplasms (Figure 4A (a1 and a2)), while A549 cells treated with denatonium showed large amplitude swelling of mitochondria (Figure 4A (b1, b2 and b3)) (2 mM for 72 h).

### Denatonium decreases mitochondrial membrane potential

Loss of mitochondrial membrane potential is a sign of the apoptotic process in cells. To further confirm mitochondrial damage, we stained airway epithelial cells with the cationic lipophilic dye JC-1. JC-1 accumulates in healthy mitochondria as aggregates with red fluorescence, while in depolarized or damaged mitochondria, JC-1 transforms into monomers with green fluorescence. As shown in Figure 4 and Additional file 3: Figure S3, denatonium treatment of airway epithelial cells resulted in a dose-dependent increase in the percentage of green-fluorescent-positive cells, which are shown in the right (R) quadrant of the fluorescence activated cell sorter (FACS) histogram. The mitochondrial membrane potential was reduced substantially (drastic increase in JC-1 monomer) in airway epithelial cells when they were treated with 2 mM denatonium.

### Denatonium down-regulates the expression of the anti-apoptotic protein Bcl-2 and enhances the release of cytochrome c and Smac/DIABLO from mitochondria in 16HBE cells

**Figure 5:**
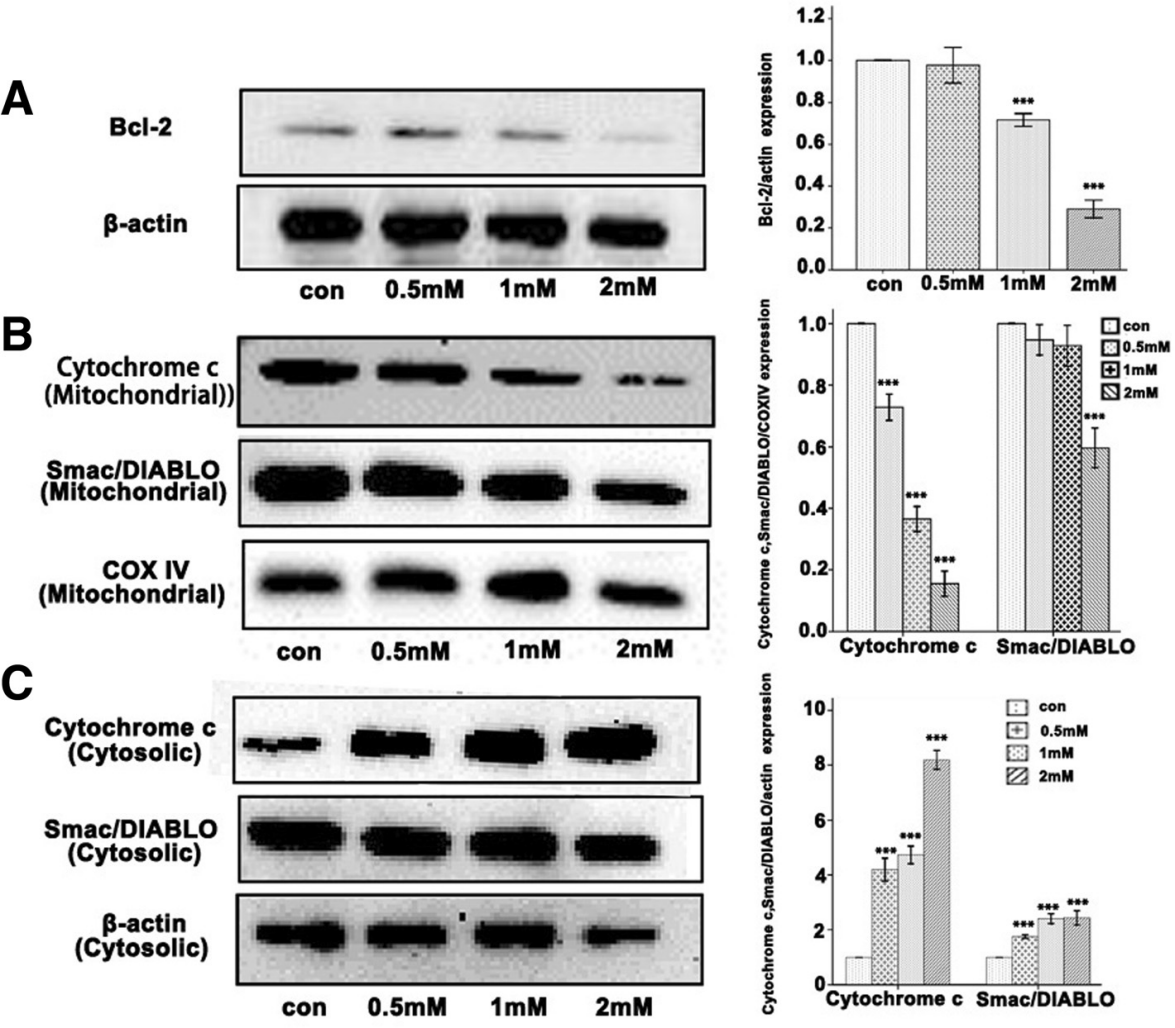
**Denatonium down-regulates the expression of the anti-apoptotic protein Bcl-2 and enhances the release of cytochrome c and Smac/DIABLO from mitochondria in 16HBE cells. A)** The expression of the anti-apoptotic protein Bcl-2 was significantly reduced in 16HBE cells after treatment with denatonium for 24 h. **B)** In 16HBE cells treated with denatonium for 24 h, the cytochrome c and Smac/DIABLO levels in mitochondria were significantly reduced, whereas, the cytochrome c and Smac/DIABLO levels in cytosol were significantly increased **(C)**. The figures are representative profiles of at least three experiments. (***p < 0.001).

To investigate the molecular mechanism underlying denatonium-induced airway epithelial cell apoptosis, we used western blotting techniques to explore the influence of denatonium on the expression of Bcl-2 protein and mitochondrial-related proteins. After treatment with 2 mM denatonium for 24 h, the expression of the anti-apoptotic protein Bcl-2 was significantly reduced (Figure 5A), and the release of cytochrome c and Smac/DIABLO from the mitochondria to the cytoplasm was drastically increased in 16HBE cells (Figure 5B and C).

## Discussion

Denatonium, one of the most bitter known substances, has been widely used as a bitter taste agonist and has many functions in various organs. In this study, we found that denatonium inhibits airway epithelial cell proliferation, reduces the number of cells in S phase and increases cell apoptosis in a dose-dependent manner via a mitochondrial signaling pathway. For the first time, we demonstrated that denatonium decreases mitochondrial membrane potential and induces mitochondrial damage in airway epithelial cells.

For decades, millimolar doses of denatonium have been used in most studies. For example, 1–2.5 mM denatonium has been commonly used to induce bitter taste [1,22,23], to relax smooth muscle cells [7,15], and to study the beating of cilia on airway epithelial cells [5]. In this study, we tested the effects of 0.5-2 mM denatonium on A549, 16HBE, and BEAS-2B cells and found that denatonium induced apoptosis in all three of these airway epithelial cell lines. The three cell lines had different sensitivities to denatonium, Approximately 30% apoptosis was observed in A549 cells after 2 mM denatonium exposure for 72 h (Figure 3A), in 16HBE cells after 1 mM denatonium exposure for 24 h (Figure 3B), and in BEAS-2B cells after 2 mM denatonium exposure for 48 h (Additional file 2: Figure S2A).

Mitochondria play critical roles in multiple cellular functions including 1) generation of ATP to supply cellular energy, 2) storage of intracellular free Ca^2+^, and 3) release of apoptotic factors, such as cytochrome c and Smac/DIABLO, which initiate nuclear death [17-20]. In this study, we found that denatonium caused mitochondrial damage as indicated by mitochondrial swelling and the loss of mitochondrial membrane potential after denatonium treatment. The loss of mitochondrial membrane potential opens the mitochondrial permeability transition pores resulting in the release of cytochrome c and Smac/DIABLO from the mitochondria. Furthermore, we found that denatonium down-regulated Bcl-2 protein expression, which has been demonstrated to prevent the release of cytochrome c from the mitochondria [17-20]. Decreased mitochondrial function is one of the key events in apoptotic death. Thus, it is not surprising that denatonium induces the inhibition of airway epithelial cell proliferation, increases apoptotic cell death, and reduces the number of cells in S phase of the cell cycle.

In recent years, bitter taste receptors have been found to be expressed in an increasing number of organ systems and to mediate a variety of functions [2-12]. However, it is challenging to study the bitter taste receptors and their downstream signaling pathways for several reasons. First, the human genome contains ~25 TAS2R genes, several members of which are expressed on human cells to detect bitter compounds [5]; thus, it is difficult to block all these receptors using molecular techniques, such as RNA interference. Secondly, bitter agonists can activate at least four cellular mechanisms that increase intracellular Ca^2+^ levels, including 1) the TAS2R/β,γ-gustducin/PLCβ2/IP3 pathway [4-6]; 2) the TAS2R/α-gustducin/cyclic nucleotide monophosphate pathway [16]; 3) direct potassium channel inhibition [14]; and 4) direct nonselective cation channel activation [20]. Thus, it is also difficult to block all the downstream signaling pathways of bitter agonists. Thirdly, although several studies have reported inhibitory effects of probenecid [17] and amino acid derivatives [19] on bitter taste receptors, no widely tested bitter taste receptor antagonists are available. Thus, our current strategy to study the bitter taste receptors and their downstream signaling pathways was to use multiple bitter taste agonists, such as denatonium, chloroquine, saccharin, colchicine, quinine, salicin, strychnine and yohimbine. However, almost every bitter agonist commonly used has side effects: for example, chloroquine is an antimalarial, a potent autophagic drug, that inhibits Na^+^,K^+^-ATPase activity [18]. As denatonium [1,2,12,22-28] and chloroquine [29] have both been used as the sole bitter agonist in many studies, denatonium- and chloroquine-induced effects as bitter taste receptor activation should be interpreted with caution.

## Conclusions

For the first time, we demonstrated that denatonium inhibits airway epithelial cell proliferation, reduces the number of cells in S phase and increases cell apoptosis in a dose-dependent manner via a mitochondrial signaling pathway. Denatonium plays critical roles in taste detection, neurotransmitter release, smooth muscle relaxation, and innate immunity, and could be potentially used for asthma treatment; however, the underlying mechanism of action of denatonium is unknown. We studied the effects of denatonium on mitochondria and revealed a novel mitochondrial-related signaling pathway.

## Additional files

Additional file 1: Figure S1. Denatonium inhibits BEAS-2B cell proliferation and induces cell morphological changes. A) Bright-field images of cultured BEAS-2B cells showed that treatment with denatonium for 48 h induced cell morphological changes. One representative experiment with n = 3 is shown. B) Denatonium markedly inhibited the growth of BEAS-2B cells in a dose-dependent manner at 48 h. The error bars represent mean values ± SEM. *** indicates significant difference at *p* < 0.001 versus control. Additional file 2: Figure S2. Flow cytometric analysis of apoptosis induction and cell cycle distribution in BEAS-2B cells. A) BEAS-2B cells were treated with denatonium (0.5 mM, 1 mM or 2 mM) for 48 h, stained with FITC-annexin V/PI and then analyzed by flow cytometry. The right panel shows the apoptosis rates of the cells in the various groups. B) Flow cytometry was used to analyze DNA in cells in the G1, S, and G2 phases of the cell cycle. Data are representative of three similar experiments.(**P < 0.01, ***P < 0.001). Additional file 3: Figure S3. Denatonium reduces mitochondrial membrane potential. A) We stained BEAS-2B cells with the cationic lipophilic dye JC-1 and analyzed them by FACS. The figures are representative profiles of at least three experiments. B) The histogram shows the quantification of mitochondrial membrane potential. (**P < 0.01 and ***P < 0.001).

## Abbreviations

DMEM: Dulbecco’s modified Eagle medium
TAS2R4: Taste receptor type 2 member 4
TAS2R10: Taste receptor type 2 member 10
GNAT3: Guanine nucleotide-binding protein G(t) subunit alpha-3
TRPM5: Transient receptor potential cation channel subfamily M member 5
CCK-8: Cell Counting Kit-8
TEM: Transmission Electron Microscopy
