# Supplementary figures and images for: Denatonium inhibits growth and induces apoptosis of airway epithelial cells through mitochondrial signaling pathways
Source: Respir Res. 2015 Feb 5;16(1):13. doi: 10.1186/s12931-015-0183-9 (PMC4326484; doi:10.1186/s12931-015-0183-9)

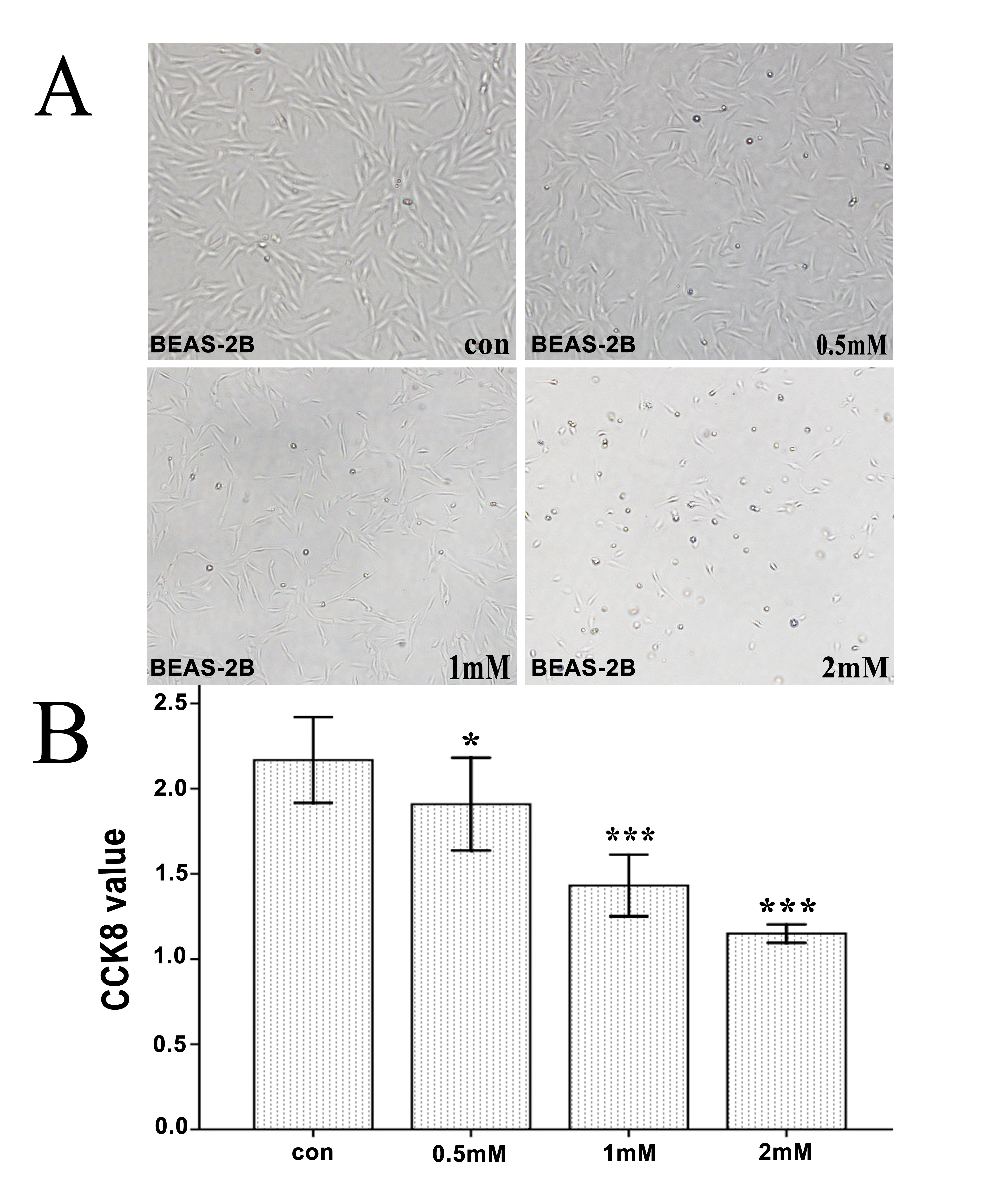

Supplement: Additional file 1: Figure S1. — Denatonium inhibits BEAS-2B cell proliferation and induces cell morphological changes. A) Bright-field images of cultured BEAS-2B cells showed that treatment with denatonium for 48 h induced cell morphological changes. One representative experiment with n = 3 is shown. B) Denatonium markedly inhibited the growth of BEAS-2B cells in a dose-dependent manner at 48 h. The error bars represent mean values ± SEM. *** indicates significant difference at p < 0.001 versus control. [file 12931_2015_183_MOESM1_ESM.jpeg]

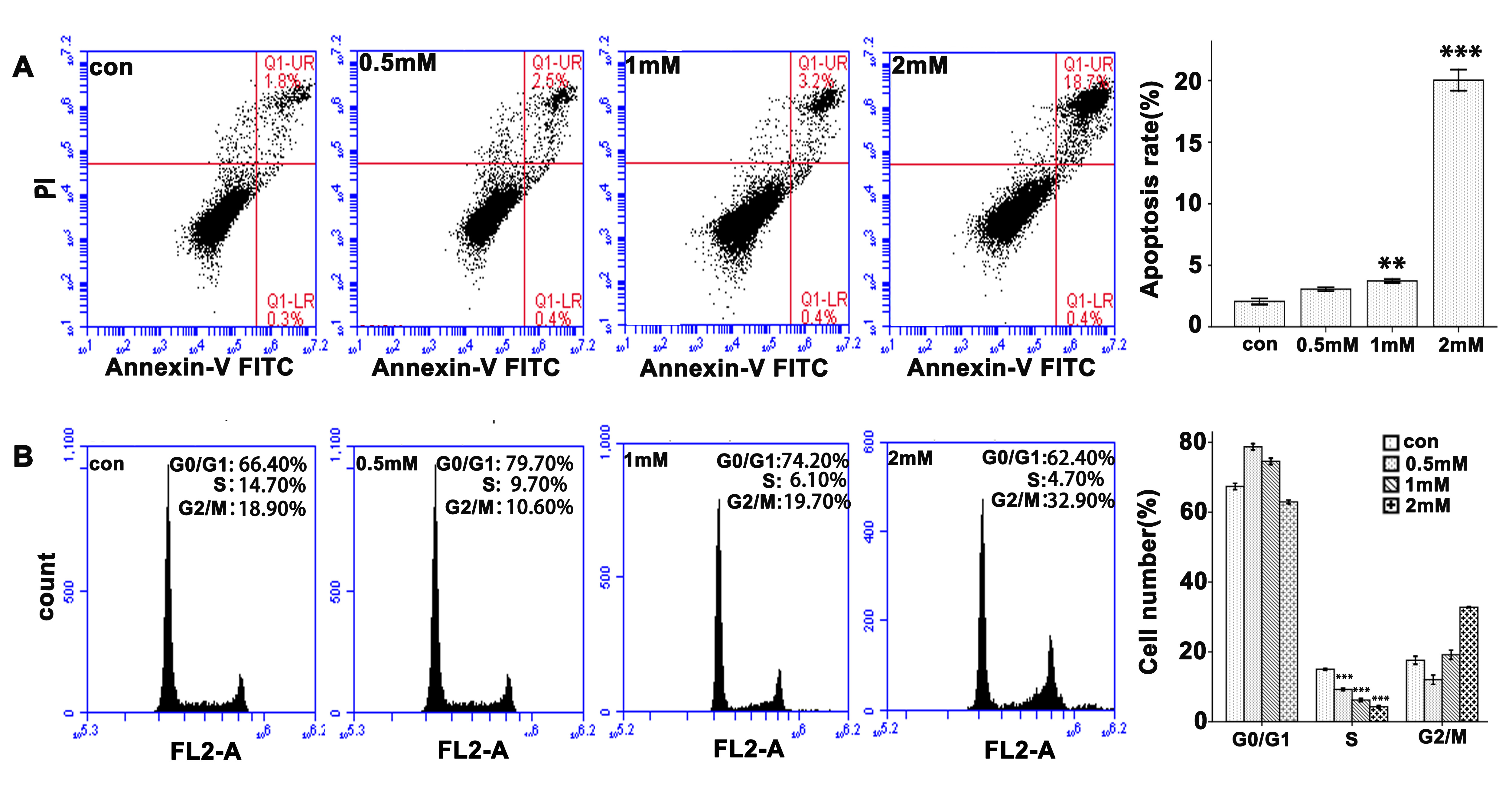

Supplement: Additional file 2: Figure S2. — Flow cytometric analysis of apoptosis induction and cell cycle distribution in BEAS-2B cells. A) BEAS-2B cells were treated with denatonium (0.5 mM, 1 mM or 2 mM) for 48 h, stained with FITC-annexin V/PI and then analyzed by flow cytometry. The right panel shows the apoptosis rates of the cells in the various groups. B) Flow cytometry was used to analyze DNA in cells in the G1, S, and G2 phases of the cell cycle. Data are representative of three similar experiments.(**P < 0.01, ***P < 0.001). [file 12931_2015_183_MOESM2_ESM.jpeg]

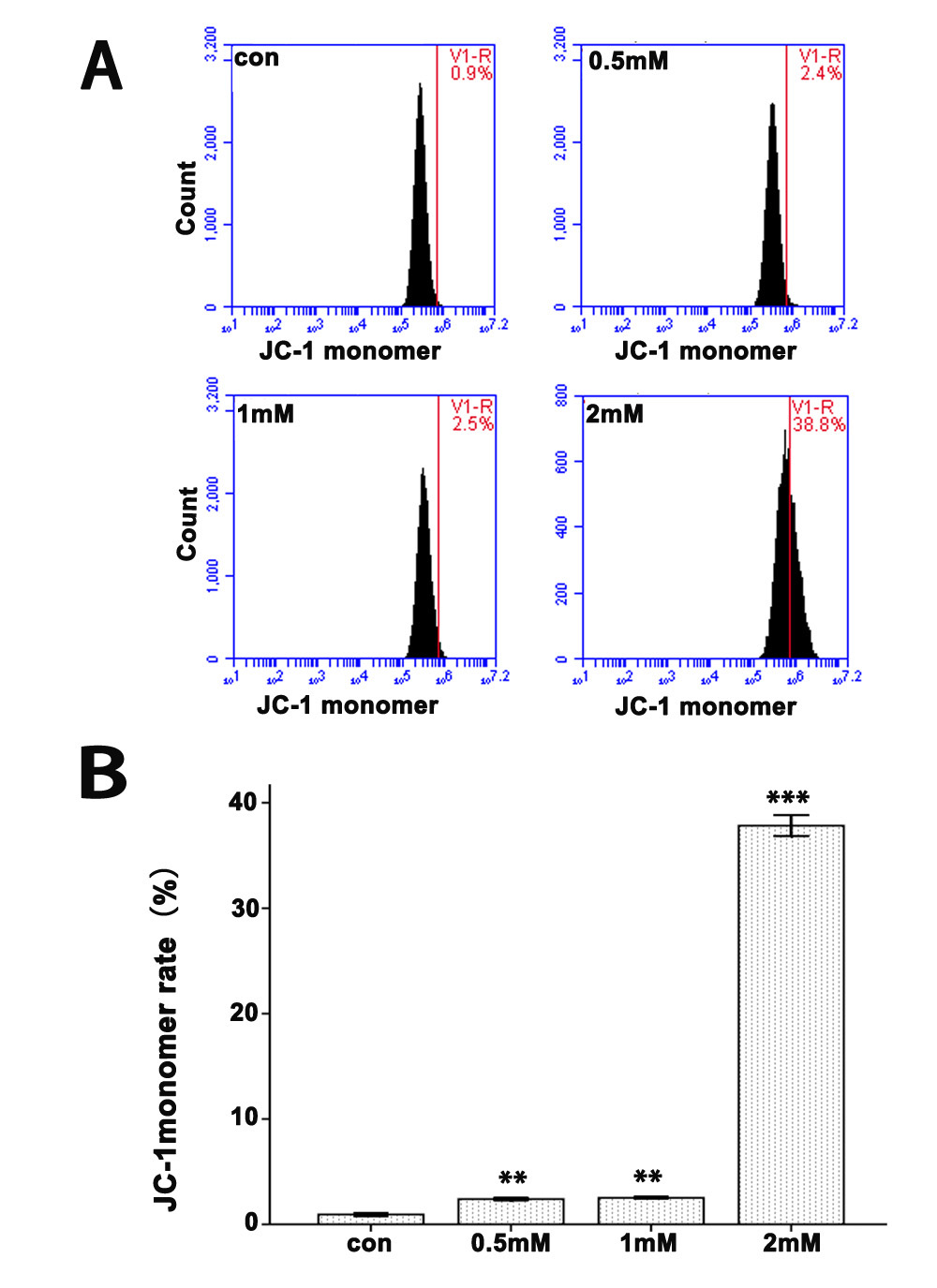

Supplement: Additional file 3: Figure S3. — Denatonium reduces mitochondrial membrane potential. A) We stained BEAS-2B cells with the cationic lipophilic dye JC-1 and analyzed them by FACS. The figures are representative profiles of at least three experiments. B) The histogram shows the quantification of mitochondrial membrane potential. (**P < 0.01 and ***P < 0.001). [file 12931_2015_183_MOESM3_ESM.jpeg]
